# Offensive Breath

**Published:** 1872-06

**Authors:** H. V. Kaggey

**Affiliations:** Dentist, Areola, Ill


### Offensive Breath.

BY H. V. KAGGEY, DENTIST, AREOLA, ILL.

The Scriptures say, " God breathed into man and he became a living soul. But now-a-days, to judge of some men's breath, we would be induced to conclude they had any thing else but a *living* soul within.

How often do we meet individuals, friends, whom otherwise we would be pleased to greet, but from whom the formal "how do you do?" alone is sufficient to turn our heads aside in disgust! How often in traveling, in public assemblies, in the social circle, do we come in contact with persons whose own exhalations are enough to throw us into a state of asphixia, and here, as it were, get us dreaming of tombs, of devouring worms, of putrefaction, of total oblivion! Peradventure you smile at the dreamer's reverie; but, in the name of decency, has the picture been overdrawn? Did you wear a tranquil, smiling countenance the other Sabbath, when crowded into your pew—retreat cut off—and you stood beside that fellow whose breath troubled your stomach, as do the winds the waters of the deep? Echo answers no. It is to be regretted that many persons of refinement have asked their most frank and confidental friends, "Is my breath bad?" and nine times out of ten the negative answer given has been *false*.

Alas! when will man learn to be just to his fellow man? "Truth loves open dealing." By the mild phrase, "bad breath," is simply implied all that is disagreeable, obnoxious and repulsive, and arises from the result of some physical derangement; most generally attributable to either the lungs, stomach or mouth.

But since the first two of these, on account of location, are difficult to diagnose and treat (and other reasons herein given), we would advise, first, an examination of the mouth; seek the advice and services of some dentist of known ability and respect—not a cosmopolite. And if the mouth with its indwelling organs and parts is unimpaired and healthy, then, as thoroughly as possible, inquire into the hidden mysteries of the other two, through the medium of some like respectable, worthy and reliable physician. But from the fact that the majority of cases of foul breath arise from a foul mouth, with irregular and carious teeth, ulcerated roots, fungous gums, or inflamed membranes, we think we can well assure you, if you will attend to these latter things, it will be all that is necessary; and the ultimate result will be, you will breathe a purer sweeter breath, and live and breathe it longer.

